# Genotype–Drug–Diet Interactions in Metabolic Regulation: CYP1A2 rs762551 Modulates the Effect of Caffeine on Lipid and Glucose Profiles in the Context of Pharmacotherapy

**DOI:** 10.3390/nu17142288

**Published:** 2025-07-10

**Authors:** Laura Claudia Popa, Ahmed Abu-Awwad, Simona Sorina Farcas, Simona-Alina Abu-Awwad, Nicoleta Ioana Andreescu

**Affiliations:** 1 Doctoral School, “Victor Babes” University of Medicine and Pharmacy, Eftimie Murgu Square, No. 2, 300041 Timisoara, Romania; laura.popa@umft.ro; 2 Department of Microscopic Morphology, Discipline of Genetics, Genomic Medicine Centre “Victor Babes”, University of Medicine and Pharmacy, 300041 Timisoara, Romania; farcas.simona@umft.ro (S.S.F.); andreescu.nicoleta@umft.ro (N.I.A.); 3 “Pius Brinzeu” Emergency Clinical County Hospital, Bld Liviu Rebreanu, No. 156, 300723 Timisoara, Romania; ahm.abuawwad@umft.ro; 4 Department XV—Discipline of Orthopedics—Traumatology, “Victor Babes” University of Medicine and Pharmacy, Eftimie Murgu Square, No. 2, 300041 Timisoara, Romania; 5 Research Center University Professor Doctor Teodor Sora, “Victor Babes” University of Medicine and Pharmacy, Eftimie Murgu Square, No. 2, 300041 Timisoara, Romania; 6 Department XII, Discipline of Obstetrics and Gynecology, “Victor Babes” University of Medicine and Pharmacy, Eftimie Murgu Square, No. 2, 300041 Timisoara, Romania; 7 “Louis Turcanu” Children Emergency Hospital, 300011 Timisoara, Romania

**Keywords:** blood glucose, caffeine, cholesterol, LDL, cytochrome P-450 CYP1A2, gene–environment interaction, hypoglycemic agents, hypolipidemic agents, pharmacogenetics

## Abstract

**Background/Objectives**: Inter-individual metabolic responses to caffeine are shaped by CYP1A2 clearance rate and by concurrent lipid- or glucose-lowering drugs. We investigated how habitual caffeine intake relates to serum cholesterol and fasting glucose under different CYP1A2 rs762551 genotypes and statin or oral antidiabetic (OAD) use. **Methods**: A prospective cross-sectional analysis was performed on 358 adults (AA = 65, AC = 163, CC = 130) with recorded genotype, daily caffeine intake, total cholesterol, fasting glucose, and medication status. Multivariable linear regression tested the main and interaction effects of caffeine (mg day^−1^), genotype, and therapy. **Results:** Caffeine intake was positively associated with cholesterol levels (β = 0.30; *p* < 0.001). A significant genotype × caffeine interaction (β = 0.27; *p* < 0.001) revealed the steepest rise in fast metabolisers (AA) not on statins, an effect largely blunted by statin therapy. For glucose, the genotype × caffeine term was also significant (β = 0.30; *p* < 0.001). Among slow metabolisers (CC) without OADs, caffeine correlated positively with glycaemia (r = 0.34; *p* = 0.028), whereas in fast metabolisers on OADs the association reversed (r = −0.36; *p* = 0.015). No meaningful associations occurred in AC carriers. **Conclusions**: Caffeine’s metabolic impact depends jointly on CYP1A2 genotype and medication. It raises cholesterol in fast metabolisers lacking statins and elevates glucose in slow metabolisers without OADs, yet may lower glucose when rapid metabolisation coincides with antidiabetic therapy. Accounting for this gene–diet–drug interplay could refine caffeine guidance within precision nutrition frameworks.

## 1. Introduction

Caffeine is one of the most commonly consumed substances worldwide, found not only in coffee but also in tea, soft drinks, energy beverages, and even certain medications [1]. While its acute stimulant effects on the central nervous system are well documented and widely appreciated, there is growing interest in its long-term impact on metabolic health [1]. The data, however, are inconsistent; some studies associate caffeine with favorable metabolic outcomes, such as improved lipid profiles and reduced risk of type 2 diabetes [2,3], while others suggest potentially adverse effects. These discrepancies suggest that the metabolic impact of caffeine may be highly individualized, influenced by both genetic and pharmacological contexts [4,5].

One of the key genetic players in caffeine metabolism is the cytochrome P450 1A2 enzyme, encoded by the CYP1A2 gene [6]. A well-known single-nucleotide polymorphism, rs762551, results in individuals being classified as “fast” (AA genotype) or “slow” (AC or CC) metabolizers of caffeine [7]. This polymorphism affects the rate at which caffeine is cleared from the body, potentially altering both its physiological and metabolic effects [7].

Modern patients, especially those with components of metabolic syndrome, are frequently treated with medications such as statins for dyslipidemia or oral antidiabetic (OAD) drugs for glycemic control [8]. These therapies are not metabolically inert. On the contrary, they actively shape lipid and glucose homeostasis and may either obscure or exaggerate the effects of dietary compounds like caffeine [9,10]. This raises a crucial but underexplored question: To what extent do medications modulate the genetic impact of caffeine on metabolic parameters?

There is some evidence suggesting that the association between caffeine and lipid levels may be blunted in patients on statin therapy, implying a potential pharmacological “masking effect.” Similarly, caffeine’s interaction with glucose metabolism might be altered in individuals receiving antidiabetic treatment [11]. This triad (caffeine intake, genotype, and concurrent medication) forms a complex interplay that has been largely overlooked in both clinical studies and nutritional guidelines.

The present study examines how habitual caffeine consumption modulates two key metabolic outcomes (total cholesterol and fasting glucose) within the nutritional context of differing CYP1A2 rs762551 genotypes and concurrent pharmacotherapy (statins or oral antidiabetics). Leveraging a well-characterized cohort with integrated dietary, genetic, and medication data, we test the hypothesis that caffeine’s metabolic effects are genotype- and drug-dependent rather than uniform across individuals. By embedding gene–diet–drug interactions into the analysis, the work advances the field of precision nutrition and offers a clinically realistic framework for personalized dietary guidance.

## 2. Materials and Methods

### 2.1. Study Design

This was a cross-sectional observational study with hybrid data collection: clinical and laboratory data were retrieved retrospectively from medical records, while dietary caffeine intake was prospectively collected via structured questionnaires. This study was designed to investigate the interplay between genetic variation in caffeine metabolism (specifically the CYP1A2 rs762551 polymorphism), dietary caffeine intake, and metabolic outcomes—namely serum cholesterol and fasting glucose levels—in adult patients with or without pharmacologic treatment for dyslipidemia or type 2 diabetes.

The study lot includes both male and female participants, aged 18 and above. Eligible individuals had available genotyping results for the CYP1A2 rs762551 locus, documented daily caffeine intake, and recent laboratory measurements of lipid and glucose profiles. Patients were further categorized based on the use of statins and OAD medications at the time of data collection.

Participants were stratified into three genotype groups and within each genotype category, we examined the association between self-reported caffeine intake (mg/day) and metabolic parameters, with subgroup analyses based on the presence or absence of statin and/or OAD therapy.

This study was conducted in accordance with the principles of the Declaration of Helsinki and received ethical approval from the University of Medicine and Pharmacy “Victor Babes” from Timisoara (No. 86/2020). All patient data were deidentified before analysis to ensure confidentiality.

### 2.2. Participants

This study enrolled 358 adults whose records contained complete data on genetic background, daily caffeine intake, current medications, and key metabolic markers. All had previously been genotyped for the CYP1A2 rs762551 polymorphism and had recent fasting total cholesterol and plasma glucose measurements. Eligibility required age ≥ 18 years, a documented CYP1A2 genotype (AA, AC, or CC), and an average daily caffeine intake reported through structured dietary questionnaires. Up-to-date values for serum cholesterol and fasting plasma glucose (within the previous six months) and precise documentation of statin and/or oral antidiabetic (OAD) use were also required. Another inclusion criterion was a self-reported history of regular caffeine consumption (minimum 20 years), with coffee being the primary source. This ensured that the analysis reflected long-term exposure rather than acute or occasional consumption.

Exclusion criteria comprised missing data, pregnancy or breastfeeding, active cancer treatment, major untreated endocrine disorders (e.g., thyroid disease or Cushing’s syndrome), and extreme caffeine consumption (>600 mg/day). Participants were grouped by genotype—AA (*n* = 65), AC (*n* = 163), and CC (*n* = 130)—and further stratified according to concomitant statin or OAD therapy to facilitate analysis of gene–diet–drug interactions. All data were collected retrospectively and anonymized before analysis; no interventions were applied.

### 2.3. Variables and Data Collection

Daily caffeine intake (mg/day) was the principal exposure variable in this study. Intake estimates were based on individually reported sources of caffeine, including coffee, tea, energy drinks, soft drinks, and caffeine-containing medications. Participants provided data through a structured questionnaire and four non-consecutive 24 h dietary recalls, conducted by trained personnel using the validated five-pass method. During these assessments, participants reported the volume and type of coffee consumed daily (e.g., caffeinated or decaffeinated), along with any added ingredients such as milk and sugar.

Although the specific sources of caffeine were recorded individually, the high prevalence of mixed consumption patterns (e.g., coffee and tea on the same day) precluded meaningful stratification by beverage type. Therefore, total daily caffeine intake (mg/day) was used as the main exposure. The reported servings were converted into standardized caffeine values using reference amounts (e.g., 95 mg per cup of brewed coffee, 47 mg per cup of black tea), and final estimates were calculated using the Nutritio web application, which automatically translated portion sizes into daily nutrient and caffeine content.

The CYP1A2 rs762551 genotype, determined by validated laboratory procedures, was recoded into three metabolic phenotypes—fast (AA), intermediate (AC), and slow (CC) caffeine metabolizers.

Primary outcomes were total serum cholesterol and fasting plasma glucose, both expressed in mg/dL and obtained within the previous six months under confirmed fasting conditions.

Additional covariates included age, sex, obesity status (BMI ≥ 30 kg/m^2^) [12], and physician-documented comorbidities such as type 2 diabetes, hypertension, and hepatic steatosis. All variables were reviewed for completeness and internal consistency before inclusion in the final analysis.

### 2.4. Genotyping

Genomic DNA was extracted from peripheral blood samples using the MagCore Nucleic Acid Extraction Kit (RBC Bioscience, New Taipei City, Taiwan), following the standard instructions provided by the manufacturer. Our data demonstrate that an identical caffeine dose can elicit sharply different outcomes. The concentration and purity of the extracted DNA were then assessed using an Epoch Microplate Spectrophotometer (Agilent BioTek, Santa Clara, CA, USA) to ensure the quality of samples prior to analysis.

Genotyping was performed using a TaqMan Real-Time PCR assay (Assay ID C_8881221) with TaqMan™ Genotyping Master Mix (Thermo Fisher Scientific, Waltham, MA, USA). All procedural steps strictly adhered to the manufacturer’s protocol as described in the Applied Biosystems TaqMan SNP Genotyping Assays documentation.

Amplification reactions were run on the LightCycler 480 Real-Time PCR System (Roche Diagnostics, Basel, Switzerland), and genotype discrimination was performed using Gene Scanning software, version 1.5.1. Each reaction was carried out in a 96-well plate, with a consistent volume of 20 µL to ensure uniformity across samples.

The TaqMan assay used in this protocol included two allele-specific probes, each tagged with a different fluorescent dye to distinguish between wild-type and variant alleles. Following PCR amplification, allele calling was based on endpoint fluorescence measurements captured directly by the system.

To maintain analytical rigor and minimize bias, genotype assessment was performed independently by two researchers blinded to sample identity. Additionally, a random subset of 5% of the samples was re-tested to verify reproducibility and ensure genotyping accuracy.

### 2.5. Statistical Analysis

Statistical analysis in this study was conducted using a combination of GraphPad Prism version 6.0 and MedCalc version 20.0, chosen for their reliability in clinical research and flexibility in handling subgroup and correlation-based analyses. Before any formal comparisons were made, we assessed the distribution of continuous variables using the Shapiro–Wilk test and visual inspection of histograms to confirm normality. Most metabolic variables followed a normal distribution and were therefore described using mean and standard deviation, while categorical data were summarized as absolute numbers and percentages.

To explore how baseline clinical and demographic variables differed across genotype groups (AA, AC, and CC), we used one-way ANOVA for continuous outcomes and chi-square or Fisher’s exact tests for categorical variables, depending on cell sizes. Where significant differences emerged, we applied Bonferroni-adjusted post hoc tests to pinpoint specific group-level differences.

The core of our analysis focused on understanding how caffeine intake relates to metabolic markers—specifically total cholesterol and fasting glucose—and how these relationships are influenced by both genetic background and pharmacologic treatment. For this purpose, we used Pearson correlation coefficients within each genotype subgroup, stratifying the cohort further by treatment status (statin or OAD use). This approach allowed us to detect genotype-specific associations that would have been masked in an aggregated dataset.

To better understand the interactive effects of genetics, diet, and pharmacotherapy, we built two multivariable linear regression models—one for cholesterol and one for glucose levels. These models included genotype, caffeine intake, and treatment status as independent variables, and critically, we incorporated interaction terms (genotype × caffeine and treatment × caffeine) to explicitly test for moderation effects. All models were adjusted for clinically relevant covariates such as age, sex, and obesity status when applicable.

In addition to numerical outputs, we emphasized graphical data visualization throughout the analysis. Scatterplots, interaction line graphs, and comparative boxplots were generated to illustrate trends and clarify how caffeine’s metabolic effects differed across genotypes and treatment subgroups. These visual tools were especially helpful in communicating the direction and strength of associations in a more intuitive, reader-friendly manner.

Throughout all analyses, a two-tailed *p*-value < 0.05 was considered statistically significant.

## 3. Results

Before exploring the interaction effects between genotype, pharmacological treatment, and caffeine intake on metabolic outcomes, we examined the baseline characteristics of the study cohort stratified by CYP1A2 rs762551 genotype. As shown in Table 1, the three genotype groups (AA, AC, and CC) displayed broadly similar demographic and clinical profiles. There were no statistically significant differences in sex distribution, age, or average caffeine consumption across genotypes. Likewise, metabolic parameters such as cholesterol, fasting glucose, and the prevalence of obesity, type II diabetes mellitus, hypertension, and hepatic steatosis showed no meaningful divergence between groups. These findings suggest a relatively balanced baseline, supporting the assumption that any subsequent differences in metabolic outcomes are more likely to reflect genuine gene–treatment–diet interactions rather than underlying demographic or clinical confounders.

To further investigate the interplay between caffeine metabolism and lipid profiles in relation to CYP1A2 genotype and pharmacological status, the cohort was stratified based on statin use. As summarized in Table 2, genotype-specific patterns in cholesterol response to caffeine intake emerged, particularly among individuals not receiving statin therapy. In this subgroup, a significant positive correlation was observed between caffeine intake and cholesterol levels in AA carriers, with a moderate association also present in those with the CC genotype. In contrast, the AC genotype appeared metabolically neutral in this context. Interestingly, in statin users, these associations were markedly attenuated or lost altogether, with correlation coefficients approaching zero and no statistically significant relationships detected. These findings suggest a potential masking effect of statin therapy on genotype-dependent lipid responses to caffeine and reinforce the hypothesis that pharmacological treatment may modulate gene–diet interactions in clinically relevant ways.

To explore the relationship between caffeine intake and glucose homeostasis across genetic backgrounds, participants were stratified by the use of OAD drugs. As presented in Table 3, among individuals not receiving glucose-lowering therapy, a significant positive correlation was identified between caffeine intake and fasting glucose levels in CC genotype carriers, suggesting a potential adverse metabolic response in slow metabolizers. Conversely, this association was absent in AA and AC subgroups, indicating a possible genotype-specific sensitivity to caffeine. In contrast, among OAD users, the pattern shifted notably: while most correlations remained non-significant, AA genotype carriers exhibited a statistically significant inverse relationship between caffeine intake and glucose levels. This suggests a potential beneficial interaction between caffeine and antidiabetic treatment in fast metabolizers. Collectively, these findings underscore the importance of considering both pharmacological context and genetic predisposition when evaluating dietary influences on glycemic control.

To synthesize the complex interrelationships between genotype, pharmacological therapy, and caffeine intake, we constructed an integrative model highlighting clinically relevant interactions on metabolic outcomes. As outlined in Table 4, distinct genotype–treatment combinations revealed the significant modulation of caffeine’s effects on both lipid and glucose metabolism. Among individuals with the AA genotype, caffeine intake was positively associated with elevated cholesterol levels in those not on statin therapy, while the same genotype showed an inverse relationship with glucose in the context of antidiabetic treatment, suggesting a potential therapeutic synergy. In contrast, CC genotype carriers not receiving glucose-lowering therapy exhibited a positive association between caffeine and glycemia, pointing to a possible risk profile in slow metabolizers. Notably, the AC genotype showed no significant interactions across treatment contexts, potentially reflecting a metabolically neutral phenotype. These findings support the presence of nuanced, genotype-specific dietary effects that are either revealed or attenuated depending on concurrent pharmacologic interventions.

**Figure 1:**
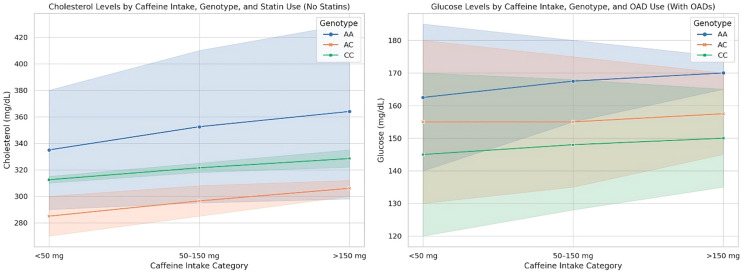
Differential metabolic responses to caffeine by genotype and treatment group.

To visualize the interaction between CYP1A2 genotype, caffeine intake, and pharmacological status on key metabolic outcomes, we constructed stratified interaction plots, presented in Figure 1. These graphs illustrate how variations in caffeine consumption translate differently into cholesterol and glucose responses depending on both genetic background and ongoing treatment. Notably, the trajectories of the lines reveal divergent patterns across genotypes, with the effect of caffeine appearing more pronounced in certain subgroups and blunted in others, particularly in the context of pharmacological modulation. This graphical representation complements the statistical models by offering a clear, intuitive snapshot of how metabolic responses to a common dietary component are not uniform, but shaped by the combined influence of inherent genetic variation and treatment context.

**Figure 2:**
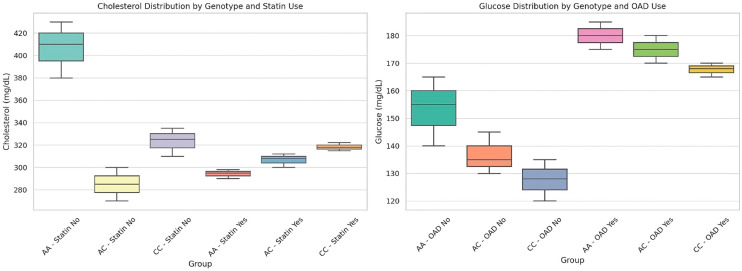
Distribution of cholesterol and glucose levels by genotype and pharmacological treatment.

To provide a more nuanced perspective on the distributional differences across genotypes and treatment groups, we generated comparative boxplots of cholesterol and glucose values, shown in Figure 2. This visual format highlights the variability within each subgroup, while emphasizing shifts in central tendency and dispersion driven by both genetic background and pharmacologic context. The data reveal distinct clustering patterns, suggesting that genotype–treatment combinations influence not only mean metabolic levels but also the degree of interindividual variability. Outliers and interquartile ranges further illustrate how some subpopulations are more metabolically heterogeneous than others, reinforcing the importance of personalized approaches in interpreting common biomarkers like cholesterol and glucose.

**Figure 3:**
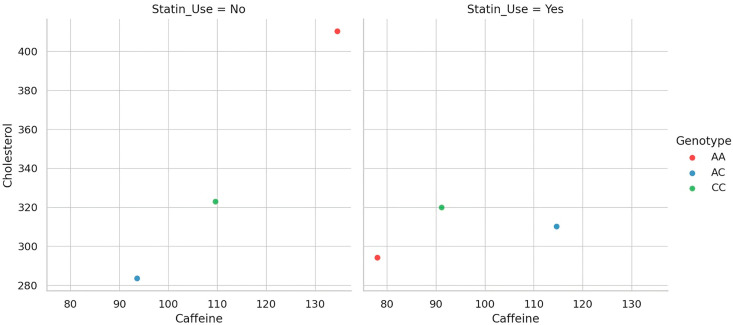
Caffeine–cholesterol associations by CYP1A2 genotype and statin use.

To better delineate the gene–diet–drug interaction underlying cholesterol metabolism, we visualized the relationship between caffeine intake and total cholesterol levels across genotype groups, separately for statin users and non-users. As depicted in Figure 3, distinct patterns emerge when stratifying the cohort by treatment status. Each dot represents an individual participant, showing the relationship between daily caffeine intake and total cholesterol, by genotype and statin use. Among non-users of statins, the association between caffeine and cholesterol appears more variable and genotype-dependent, suggesting that the genetic modulation of caffeine metabolism exerts a more visible effect in the absence of pharmacologic interference. In contrast, within the statin-treated group, the relationship is markedly attenuated, hinting at a potential masking effect of lipid-lowering therapy. This visual stratification supports the hypothesis that genotype-driven differences in metabolic response to dietary exposures may be clinically relevant only when not obscured by pharmacotherapy.

**Figure 4:**
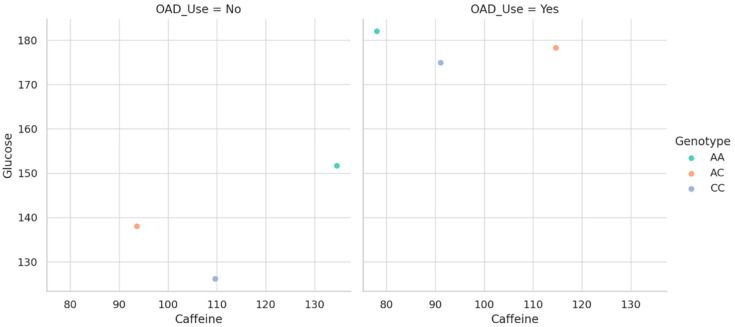
Caffeine–glucose associations by CYP1A2 genotype and OAD use.

To clarify how genetic variation modulates the glycemic response to caffeine within different pharmacologic contexts, we generated stratified scatterplots based on OAD drug use. As shown in Figure 4, the relationship between caffeine intake and fasting glucose appears to vary depending on both genotype and treatment status. Each dot corresponds to a participant, illustrating the association between caffeine intake and fasting glucose across genotype and OAD therapy groups. Among individuals not receiving antidiabetic therapy, genotype-specific trends are more distinct, highlighting a potential vulnerability in slow metabolizers when caffeine is unopposed by pharmacologic control. Conversely, in those under OAD treatment, these differences are visibly blunted, suggesting that pharmacologic intervention may buffer or override genotype-driven responses. This graphical representation underscores the value of considering gene–diet–drug interactions simultaneously, particularly in the context of glucose metabolism, where clinical management may mask or reshape inherent metabolic sensitivities.

To quantitatively assess the interactive effect of genotype and caffeine intake on glucose levels while adjusting for relevant covariates, a multivariable linear regression model was employed. As detailed in Table 5, the interaction term between genotype and caffeine intake was statistically significant, indicating that the impact of caffeine on glycemia differs meaningfully across genotypes. Specifically, the presence of a CYP1A2 AA genotype amplified the glycemic response to caffeine, even after adjusting for baseline intake and OAD use. While neither genotype nor caffeine intake alone demonstrated significant main effects in this model, the interaction term emerged as a robust predictor. Additionally, the use of OAD agents was independently associated with higher fasting glucose, likely reflecting more advanced metabolic dysregulation in this subgroup. Age was included as a continuous covariate and centered at 53 years to improve interpretability. These findings underscore the value of modeling gene–diet interactions explicitly, as their effects may be masked when analyzed in isolation.

To elucidate how caffeine influences lipid metabolism in the context of both genetic variability and pharmacological intervention, we constructed a multivariable regression model focusing on cholesterol levels as the outcome. As shown in Table 6, caffeine intake was independently associated with higher cholesterol values, and this effect was significantly modulated by genotype. Specifically, the interaction term between caffeine and the AA genotype was statistically significant, suggesting that fast metabolizers may experience a greater cholesterol-elevating effect from caffeine compared to CC carriers. Moreover, statin use emerged as a strong negative predictor of cholesterol, as expected, but also demonstrated a significant interaction with caffeine intake. This negative interaction indicates that statin therapy may attenuate the lipid-raising effect of caffeine. Notably, the main effect of genotype alone was not significant, underscoring that gene–environment interactions, rather than genotype per se, play a more substantial role in shaping metabolic outcomes in this context.

## 4. Discussion

Caffeine is among the most widely consumed psychoactive compounds worldwide. While its immediate effects on alertness and cognitive performance are well established, its long-term impact on metabolic health remains incompletely understood and continues to be the subject of active investigation [13,14]. Several studies have reported associations with improved lipid or glucose profiles, whereas others have identified links to elevated cholesterol or impaired insulin sensitivity [15,16]. These inconsistencies have prompted growing interest in the factors that may influence interindividual metabolic responses to caffeine, including genetic variability and pharmacological context. A key genetic determinant is the CYP1A2 rs762551 polymorphism, which differentiates fast from slow caffeine metabolizers and may partially explain variability in metabolic outcomes [7]. Pharmacologic agents such as statins or antidiabetic drugs act on overlapping metabolic pathways, which may attenuate or modify caffeine-related effects [17,18]. However, the combined influence of genotype, habitual caffeine consumption, and ongoing medication use has rarely been examined together in clinical cohorts.

In our cross-sectional sample of 358 adults, distinct genotype–treatment patterns emerged when caffeine intake was analyzed alongside pharmacologic exposure. Individuals with the AA genotype (typically associated with faster caffeine metabolism) had higher cholesterol levels, particularly among those not using statin therapy [19]. This association may reflect a more pronounced physiological response to caffeine in fast metabolizers, although potential mechanisms such as hepatic lipid synthesis or LDL particle dynamics were not measured and remain speculative [6]. In this subgroup, the link between caffeine consumption and cholesterol was not only biologically plausible but also reached statistical significance.

In contrast, participants with the AC genotype displayed lower obesity prevalence and tended to have lower cholesterol values overall. This may indicate a relatively neutral metabolic phenotype with regard to caffeine intake, consistent with the prior literature [20,21].

Among CC genotype carriers (classified as slow metabolizers [19]), a positive association between caffeine intake and fasting glucose was observed in individuals not receiving oral antidiabetic therapy. One possible explanation is that prolonged systemic caffeine exposure may affect glucose regulation via mechanisms such as adenosine receptor antagonism, which has been implicated in reduced insulin sensitivity [22]; however, such pathways were not evaluated in this study.

However, the pattern observed in AA genotype carriers who were also receiving oral antidiabetic therapy was particularly notable. Interestingly, within this subgroup, the usual association appeared reversed: higher caffeine intake was associated with lower glucose levels. While this may reflect a pharmacologically mediated effect, potentially through mechanisms involving hepatic or peripheral pathways, such interpretations remain speculative, as no mechanistic markers were assessed. This observation aligns with previous findings reporting genotype-dependent variability in caffeine’s metabolic effects [23], and extends this concept by incorporating pharmacologic treatment as an additional modifying factor. In doing so, the present study provides an exploratory perspective on how genetic variation, dietary exposures, and medication status may jointly shape metabolic outcomes.

From a pathophysiological perspective, some of the observed patterns may reflect interindividual differences in metabolic response shaped by both genetic background and treatment status. In fast caffeine metabolizers (AA genotype) [24] who were not on statin therapy, we found a consistent positive association between caffeine intake and cholesterol levels. This relationship may be linked to enhanced metabolic stimulation due to rapid caffeine clearance, such as catecholamine release, lipolysis, or hepatic VLDL synthesis [25]—although these mechanisms remain hypothetical and unmeasured in this study. Clinically, such associations suggest that caffeine may have differential effects in individuals with specific genetic backgrounds and untreated dyslipidemia.

Importantly, the prevalence of obesity did not significantly differ between genotype groups, suggesting that obesity is unlikely to have confounded these associations. Obesity status was also included as a covariate in our regression models to adjust for potential residual effects.

Among slow metabolizers (CC genotype) [19], a positive association between caffeine intake and fasting glucose was identified in participants not using glucose-lowering therapy. This is consistent with experimental data suggesting that prolonged caffeine exposure may impair insulin sensitivity and hinder glucose uptake in skeletal muscle [26]. However, no corresponding association with cholesterol was observed in this group, potentially indicating that caffeine’s metabolic effects in slow metabolizers may be more pronounced in glucose regulation. This hypothesis should be explored further in studies that incorporate metabolic biomarkers and adjust for dietary and behavioral confounders.

The inverse association between caffeine intake and glucose levels observed in AA genotype individuals undergoing antidiabetic therapy may indicate that pharmacologic interventions, such as metformin or SGLT2 inhibitors, could attenuate caffeine-related effects on glycemia [27]. This hypothesis is biologically plausible but remains unproven in the absence of mechanistic data, and should be interpreted with caution.

A central strength of this study is its integrative approach, combining genetic, dietary, and pharmacologic variables to explore gene–diet–drug interactions in a real-world setting. While previous studies have examined caffeine intake or CYP1A2 polymorphisms in isolation [28,29,30], our work reflects the complexity of clinical reality, where patients are simultaneously exposed to multiple metabolic influences.

Among our most notable findings is the identification of a statistically significant three-way interaction between genotype, caffeine intake, and pharmacologic therapy. This interaction revealed nuanced, context-specific associations that would likely have been missed in a more aggregated analysis.

While these findings are exploratory, they may inform future research aimed at refining personalized dietary recommendations. Current caffeine intake guidelines generally do not account for interindividual differences in metabolism or treatment context. Our results suggest that identical caffeine exposures can be associated with markedly different metabolic outcomes depending on genotype and medication use. However, any clinical or policy-level guidance must await replication in larger, prospective, and mechanistically informed studies.

In this regard, the present study provides a foundational framework for future precision-nutrition research, emphasizing the value of integrating genetic data and pharmacologic context when assessing dietary exposures.

## 5. Strengths, Limitations, and Future Directions

This study has several notable strengths. By integrating genetic, dietary, and pharmacologic data, we were able to examine interactions that are often studied in isolation but rarely modeled together in a way that reflects clinical reality. The stratified analyses based on medication use added a valuable layer of nuance, allowing us to detect subtle gene–environment interactions that might otherwise go unnoticed. Furthermore, the internal consistency observed between genotype and phenotype across multiple analyses enhances the credibility of our findings, especially given the directionality and biological plausibility of the associations we identified.

Nonetheless, certain limitations should be acknowledged. Given the cross-sectional design, we cannot infer causality or establish temporal relationships between caffeine intake and metabolic outcomes. Caffeine consumption was self-reported, which introduces the potential for recall bias or underreporting, particularly in populations with variable dietary self-assessment. Our sample was drawn from a clinical population, which may limit generalizability due to differences in comorbidity profiles and medication use compared to the broader population. The study did not adjust for lifestyle-related variables such as overall dietary quality, physical activity, or total caloric intake. These factors are known to influence both glucose and lipid metabolism and may act as unmeasured confounders in the analysis. Additionally, no detailed information was collected regarding the roast level (e.g., medium vs. dark) or preparation method (e.g., instant, filtered, or espresso) of the coffee consumed. Given that these aspects can affect the actual caffeine content per serving, their omission may have introduced measurement variability in estimating total caffeine intake. Although the sources of caffeine were recorded, no separate analyses were performed to distinguish between coffee and tea consumption, due to the frequent reporting of mixed intake. This limitation may have reduced the ability to detect source-specific metabolic effects.

Although the AA genotype group was smaller in size, a post hoc power analysis showed a statistical power of 0.99 for detecting medium-sized effects across genotype groups, suggesting that the group imbalance did not critically affect the reliability of our findings.

A further limitation concerns the pharmacological grouping strategy. All statins and all oral antidiabetic agents were treated as single homogeneous categories, without stratification by individual drug or pharmacologic subclass. This analytical choice, necessitated by sample size constraints, may have limited the ability to detect drug-specific gene–nutrient interactions, particularly given the distinct pharmacodynamic properties of agents such as rosuvastatin versus simvastatin or metformin versus SGLT2 inhibitors.

Moreover, key lifestyle-related confounders were not assessed or adjusted for in the analysis. These include tobacco use (a known inducer of CYP1A2), alcohol consumption, physical activity levels, and overall dietary quality. The absence of these variables may reduce the precision of the observed associations, particularly in the context of gene–diet–drug interaction modeling.

Another important limitation of the study is the use of the rs762551 polymorphism as the sole genetic marker for CYP1A2 activity, without accounting for environmental and epigenetic influences or validating the enzymatic phenotype through caffeine metabolite measurements.

Caffeine intake was estimated based on structured questionnaires and 24 h dietary recalls, without differentiating between filtered and unfiltered coffee or between types of energy drinks. This may introduce variability in the estimation of actual caffeine content and exposure to other bioactive compounds such as diterpenes. Additionally, intake data were not validated against objective biomarkers and may be subject to recall bias.

Looking ahead, several directions for future research are worth considering. Longitudinal studies are needed to clarify causal pathways and to better understand how caffeine interacts with genetic and pharmacologic factors over time. Mechanistic insights may be gained through functional studies, including metabolomic profiling or investigations into hepatic transporter activity, to further illuminate the biological basis of gene–drug–diet interactions. Perhaps most exciting is the prospect of randomized controlled trials testing genotype-guided dietary recommendations—particularly with respect to caffeine—in individuals with metabolic syndrome or related conditions. Such work could ultimately contribute to the development of personalized nutrition strategies, where pharmacogenetics and nutrigenomics are actively integrated into everyday clinical care.

## 6. Conclusions

The findings of this study underscore the complex and context-dependent nature of the associations between caffeine intake and metabolic outcomes when examined through the combined lens of genetic background and pharmacologic treatment. Among individuals carrying the AA genotype—characterized by faster caffeine metabolism—caffeine intake was positively associated with total cholesterol levels, particularly in those not receiving statin therapy. In contrast, among CC genotype carriers (slow metabolizers), a positive association between caffeine intake and fasting glucose was observed, but only in the absence of oral antidiabetic medication. Notably, an inverse association was found between caffeine intake and glucose levels in AA individuals undergoing antidiabetic treatment, suggesting a potential gene–drug–diet interaction.

These observations do not establish causality, given the cross-sectional design, and should be interpreted as hypothesis-generating. The associations reported here highlight the need for future prospective and interventional studies to explore whether genotype-specific metabolic responses to caffeine may have clinical relevance. Until such evidence is available, recommendations regarding caffeine intake based on CYP1A2 genotype and pharmacologic context should be made with caution.

## Figures and Tables

**Table 1:** Genotype distribution and baseline characteristics.

Variable	AA (*n* = 65)	AC (*n* = 163)	CC (*n* = 130)	*p* Value	*p* Value AA vs. AC	*p* Value AA vs. CC	*p* Value AC vs. CC
Male *	34 (52.30%)	85 (52.14%)	62 (47.69%)	0.715	>0.999	0.649	0.522
Female *	31 (47.69%)	78 (47.85%)	68 (52.30%)	0.715	>0.999	0.649	0.522
Age (years) **	53.95 ± 14.01	53.53 ± 11.54	54.28 ± 11.22	0.865	0.816	0.859	0.576
Caffeine (mg/day) **	121.21 ± 106.75	127.13 ± 102.34	119.93 ± 92.92	0.812	0.697	0.931	0.533
Cholesterol (mg/day) **	349.47 ± 231.62	294.6 ± 167.8	321.75 ± 183.06	0.400	>0.999	0.363	0.178
Glucose (mg/day) **	165.41 ± 93.55	158.67 ± 66.4	157.61 ± 54.74	0.737	0.541	0.464	0.883
Obesity *	48 (73.84%)	109 (66.87%)	101 (77.69%)	0.115	0.385	0.676	0.056
Type II Diabetes Mellitus *^(a)^	43 (66.15%)	99 (60.73%)	75 (57.69%)	0.522	0.541	0.325	0.684
Hypertension *^(a)^	42 (64.61%)	112 (68.71%)	80 (61.53%)	0.435	0.660	0.794	0.246
Hepatic Steatosis *^(a)^	16 (24.61%)	38 (23.31%)	31 (23.84%)	0.978	0.971	>0.999	>0.999
Statins *^(a)^	28 (43.07%)	66 (40.49%)	51 (39.23%)	0.875	0.834	0.718	0.921
OAD Agents *^(a)^	43 (66.15%)	99 (60.73%)	75 (57.69%)	0.522	0.541	0.325	0.684

* Percentage; ** Mean ± SD; ^(a)^ binary.

**Table 2:** Genotype-specific associations between caffeine intake and cholesterol levels in the two groups.

	Non-Users of Statins (*n* = 211)			Statin Users (*n* = 147)		
Genotype	AA (*n* = 37)	AC (*n* = 97)	CC (*n* = 79)	AA (*n* = 28)	AC (*n* = 66)	CC (*n* = 51)
Caffeine (mg/day)	106.03 ± 115.58	93.69 ± 125.58	94.18 ± 103.64	107.66 ± 130.04	114.61 ± 129.23	91.06 ± 86.94
Cholesterol (mg/dL)	393.70 ± 265.15	283.59 ± 148.64	322.92 ± 159.73	294.18 ± 170.16	310.21 ± 191.82	320.00 ± 214.88
Correlation (r)	0.45	0.07	0.29	0.28	0.01	−0.08
*p*-value	0.012	0.503	0.021	0.154	0.922	0.584

**Table 3:** Caffeine–glucose interactions modulated by genotype and therapy in a stratified cohort.

	Non-Users of OAD (*n* = 199)			OAD Users (*n* = 159)		
Genotype	AA (*n* = 22)	AC (*n* = 64)	CC (*n* = 55)	AA (*n* = 43)	AC (*n* = 99)	CC (*n* = 75)
Caffeine (mg/day)	134.99 ± 148.97	99.21 ± 133.39	109.65 ± 127.64	78.01 ± 73.77	105.31 ± 121.61	83.08 ± 72.97
Glucose (mg/dL)	151.66 ± 122.06	138.01 ± 64.83	126.28 ± 48.50	182.03 ± 48.04	178.33 ± 62.10	174.85 ± 50.05
Correlation (r)	0.22	−0.12	0.34	−0.36	0.06	−0.06
*p*-value	0.222	0.299	0.028	0.015	0.589	0.613

**Table 4:** Significant genotype × treatment × caffeine interactions on metabolic markers.

Genotype	Treatment	Caffeine (mg/day)	Effect on Cholesterol (mg/dL)	Effect on Glucose (mg/dL)	Significant Interaction
AA	No statins	134.5 ± 145.2	**↑** 410.3 ± 260.4	151.7 ± 122.1	Positive association between caffeine intake and cholesterol (r = 0.46, *p* = 0.011)
AA	OAD *	78.0 ± 73.8	294.2 ± 170.2	**↑** 182.0 ± 48.0	Inverse association between caffeine intake and glucose (r = −0.36, *p* = 0.015)
AC	No statins	93.6 ± 125.6	↓ 283.6 ± 148.6	138.0 ± 64.8	No significant interaction
AC	OAD *	114.6 ± 129.2	310.2 ± 191.8	178.3 ± 62.1	No significant interaction
CC	No statins	109.6 ± 127.6	↑ 322.9 ± 159.7	**↓** 126.2 ± 48.5	Positive association between caffeine intake and glucose (r = 0.34, *p* = 0.028)
CC	OAD *	91.1 ± 86.9	320.0 ± 214.9	174.9 ± 50.0	No significant interaction

* Oral antidiabetics; ↑ increases; ↓ decreases.

**Table 5:** Effects of genetic, dietary, and pharmacologic factors on fasting glucose levels.

Variable	β Coefficient	95% Confidence Interval	*p*-Value
Intercept (age = 53)	147.25	[133.10; 161.39]	<0.001 ^1^
Genotype (AA vs. CC)	−3.92	[−9.86; 2.01]	0.191
Caffeine intake (mg/day)	−0.05	[−0.12; 0.02]	0.144
Genotype × caffeine interaction	0.26	[0.15; 0.38]	<0.001 ^2^
OAD use (yes/no)	12.87	[3.25; 22.48]	0.009 ^3^
BMI ≥ 30 (yes/no)	10.41	[2.17; 18.66]	0.014
Age (centered at 53)	0.32	[0.10; 0.53]	0.004 ^4^

^1^ Intercept represents the estimated mean fasting glucose (mg/dL) for a 53-year-old male with CC genotype, no caffeine intake, not on OAD therapy, non-obese, and BMI < 30. ^2^ Statistically significant interaction effect between genotype and caffeine intake. ^3^ Indicates a significant positive association between OAD use and glucose levels. ^4^ Age centered at 53 years; coefficient represents the change in glucose per additional year above or below 53.

**Table 6:** Effects of genetic, dietary, and pharmacologic factors on total cholesterol levels.

Variable	β Coefficient	95% Confidence Interval	*p*-Value
Intercept	285.27	[262.59; 307.95]	<0.001 ^1^
Genotype (AA vs. CC)	2.93	[−9.69; 15.55]	0.648
Caffeine intake (mg/day)	0.30	[0.14; 0.45]	<0.001
Genotype × caffeine interaction	0.27	[0.15; 0.39]	<0.001
Statin use (yes/no)	−26.20	[−44.68; −7.72]	0.006
Statin × Caffeine interaction	−0.28	[−0.53; −0.02]	0.035

^1^ Intercept reflects estimated cholesterol for a male patient with CC genotype, no caffeine, not on statins, non-obese, and age zero (not directly interpretable).

## Data Availability

The original contributions presented in this study are included in the article. Further inquiries can be directed to the corresponding author.
